# Toward a role for the acoustic field in cells interaction

**DOI:** 10.3389/fnsys.2025.1484769

**Published:** 2025-06-18

**Authors:** Marco Girasole, Pier Francesco Moretti, Angela Di Giannatale, Virginia Di Paolo, Angela Galardi, Silvia Lampis, Simone Dinarelli, Giovanni Longo

**Affiliations:** ^1^Institute of Matter Structure, Italian National Research Council, ISM-CNR, Rome, Italy; ^2^Department of Earth System Sciences and Environmental Technologies, Consiglio Nazionale delle Ricerche, Rome, Italy; ^3^Hematology/Oncology and Cell and Gene Therapy Unit, Bambino Gesù Children's Hospital, IRCCS, Rome, Italy

**Keywords:** cell-cell interactions, nanomotion sensor, mechanical waves, acoustic field, neuroblastoma cell, cell behavior

## Abstract

Nanoscale motility of cells is a fundamental phenomenon, closely associated with biological status and response to environmental solicitations, whose investigation has disclosed new perspectives for the comprehension of cell behavior and fate. To investigate intracellular interactions, we designed an experiment to monitor movements of clusters of neuroblastoma cells (SH-SY5Y) growing on a nanomechanical oscillator (nanomotion sensor) suspended few hundreds of microns over the surface of a Petri dish where other neuroblastoma cells are freely moving. We observed that the free-to-move cells feel the presence of cells on the nearby nanosensor (at a distance of up to 300 microns) and migrate toward them, even in presence of environmental hampering factors, such as medium microflows. The interaction is bidirectional since, as evidenced by nanomotion sensing, the cells on the sensor enhance their motion when clusters of freely moving cells approach. Considering the geometry and environmental context, our observations extend beyond what can be explained by sensing of chemical trackers, suggesting the presence of other physical mechanisms. We hypothesize that the acoustic field generated by cell vibrations can have a role in the initial recognition between distant clusters. Integrating our findings with a suitable wave propagation model, we show that mechanical waves produced by cellular activity have sufficient energy to trigger mechanotransduction in target cells hundreds of microns away. This interaction can explain the observed distance-dependent patterns of cellular migration and motion alteration. Our results suggest that acoustic fields generated by cells can mediate cell-cell interaction and contribute to signaling and communication.

## Introduction

Living organisms are composite systems, often formed by a multitude of interconnected organs and components, each with its own intrinsic complexity. Downscaling in the spatial dimension of this chain, we can identify the cell as a major building block of this process. Cells can sense and respond to their environment and their interactions are essential for the proper functioning of complex organisms. This interaction happens *in vivo* through different kinds of mechanisms, including chemical sensing, *via* receptors and ion channels and mechanical sensing, through integrins and cytoskeleton, which allow cells to respond to mechanical stimulation (Ullo and Case, 2023; Dinarelli et al., 2022). These processes are crucial for various physiological functions, including development, immune response, and tissue maintenance. Among those, the most studied cellular interaction is chemical signaling. Indeed, all cells probe and sense their environment and interact with nearby cells using chemical mediators, raising interest in the metabolic pathways behind these kinds of interactions (SenGupta et al., 2022).

Recent studies have shown that, by using different artificially induced stimulations alongside chemical signaling, a second, mechanical interaction appears as an equally important pathway through which cells sense and respond to the environment (Zhou et al., 2020; Dinarelli et al., 2018b; Wuest et al., 2015; Blaber et al., 2015; Wehland et al., 2013). This is directly translated into aging pathways of red blood cells, changes in the metabolic activity of bacteria, or in the resilience of cardiomyocytes and is a key parameter in the development of cancers (Dinarelli et al., 2018b; Villalba et al., 2021; Craig and Basson, 2009; Dinarelli et al., 2018a). The ability of the cells to perform mechano-sensing and to translate such stimulation into biological patterns has led to the idea that the acoustic field may also play a role in the communication process between cells.

Sound is involved into important aspects of animal behavior and plays a crucial role in a wide range of social and ecological interactions. Sound is an essential component of the environment and fauna have adapted to use sound, developing highly specialized auditory systems to detect and interpret oscillating waves. Acoustic signals can vary in pitch, rhythm, amplitude, and are often highly structured and repeated in patterns, such as drumming or rattling (Longo et al., 2021).

Overall, sound waves can deliver, effectively and rapidly, mechanical signals produced by living systems (or can assist other forms of communication). Cells can produce and respond to mechanical waves through a medium, which propagate as an acoustic field, by involving mechanosensitive ion channels present in the cell membrane that can be sensitive to pressure change, by induction of vibrations in the extracellular matrix (ECM) transmitted to the cell through integrins and other adhesion molecules, or by influencing membrane-bound receptors and producing secondary messengers (Ambattu and Yeo, 2023). These responses are translated into micro- and nano-sized cellular movements, which are directly associated to the cellular status. Indeed, there is a strong correlation between movement and life, between energy consumption and motions or vibrations at various scales, from the level of complex organisms down to single cells, and even further to molecules and macromolecules (Alonso-Sarduy et al., 2014).

Several mechanisms are involved in cellular motility, including cytoskeleton remodeling, environmental signaling and metabolic state (Suresh and Diaz, 2021). For instance, the cell cytoskeleton, composed of actin filaments, microtubules, and intermediate filaments, typically organized into networks, can be reconfigured in response to stimuli, such as forces arising from extracellular matrix degradation, cellular remodeling and pharmacological treatment (Yanes and Rainero, 2022).

Sound has been studied for its potential in NB cell maturation or for a potential therapeutic effect in enhancing tissue regeneration (Lucas et al., 2020; Armand et al., 2025; Cho et al., 2022). The interaction of cells with an acoustic field have been exploited in oncology, exploiting the fact that healthy and cancerous cells exhibit different mechanical properties, with metastatic cells generally being more deformable than primary tumor and normal cells, probably due to alterations in their cytoskeleton (Fraldi et al., 2019; Runel et al., 2021). Theoretical models have predicted that ultrasonic vibrations may differentially affect healthy and cancerous cells, both in single-cell systems and in heterogeneous cell clusters at the mesoscale (Fraldi et al., 2016). Other experiments investigating wave-cell interactions have shown the induction of unidirectional cell migration aligned with the direction of the propagating wave, which increased at a critical wave intensity but was suppressed at higher intensities (Imashiro et al., 2021).

In this work, we investigate the role of the acoustic field in simplified yet complex, living systems such as clusters of NB cells. We designed an experiment focused on the interaction between small clusters of NB cells to study the interaction between smaller complex systems.

To this aim we selected SH-SY5Y cell line, a model in neuroscience research, which can be induced to differentiate into neuron-like cells, both cholinergic and dopaminergic, through treatments with agents such as retinoic acid (RA), Brain-Derived Neurotrophic Factor (BDNF), or cAMP (Shipley et al., 2016; Hoffmann et al., 2023). The neuronal-like differentiation is revealed by the expression of key neuronal markers, including tau protein, synaptophysin, and tyrosine hydroxylase (Lopez-Suarez et al., 2022). Additionally, they also exhibit the ability to form neurites that allow the cells to establish synaptic connections, stimulating cell-cell interactions and forming neuronal networks making them suitable for *in vitro* studies (Angiari et al., 2022; Armingol et al., 2021; Song et al., 2019; Teppola et al., 2016).

**Figure 1:**
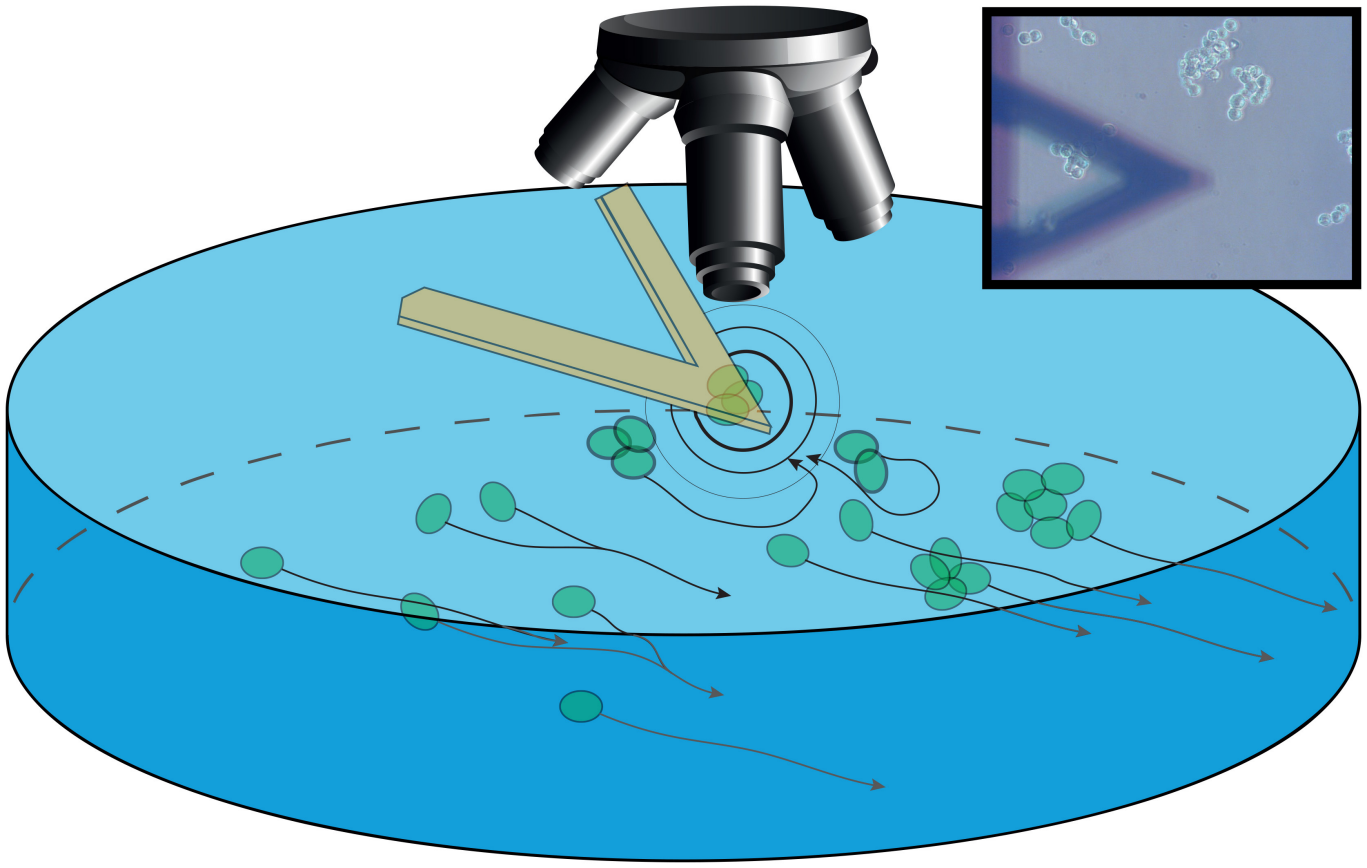
Sketch of the setup. The sensor is immersed in the growing medium in a Petri dish with an optical microscope collecting images. The sensor is bearing S-cell NB which are interacting (outward black and gray circles) with P-cells. These cells are aggregating and moving in the background following the medium flow (black arrow lines). Inset: Image of the setup as acquired through the optical microscope, with the sensor in foreground and the aggregated cells in focus on the background.

It is known that neurons in complex aggregates (such as brains) show a temporal organization of their activity, that can be represented by a system of rhythms, that has been classified for humans and for mammals in a similar way (Buzsáki et al., 2013). Currently, investigation of the neuronal activity is mainly associated by the acquisition of electric signals, while no specific correlation between their mobility and behavior of the organisms has been reported. To study NB's behavior, we coupled optical imaging with time-resolved nanoscale vibration sensors to describe cell behavior at the micro and nanoscale (Aghayee et al., 2013; Zhou et al., 2024; Longo et al., 2013; Ruggeri et al., 2017). In particular, we used the nanomotion sensor to monitor cell's vibrations as a mark of their cellular activity (Zou et al., 2023; Kasas et al., 2015; Lupoli et al., 2018; Wu et al., 2016). This particular geometrical setup, where small clusters of NB cells interact freely with other similar cells placed at a controlled distance, provided a sandbox to investigate alterations in NB activity and movement when cell-cell interactions are underway (Figure 1).

We propose that the distortion of the density field in the environment induced by the vibrations of cells adhering to the sensor can result in an unexpected contribution to communication, even at the single cell level. In fact, while it is known that cell motility is a fundamental parameter in the study of NB cells and has repercussions on the study of brain-related cancers (Ul Islam et al., 2017; Shimizu et al., 2020), we suggest that acoustic waves, such as those generated at the nanoscale by cell activity, can have an important role in the cell-cell interactions.

## Materials and methods

### Materials

DMEM low glucose, 1% penicillin-streptomycin and 1% L-Glutamin were acquired from Euroclone (Pero, MI). 10% Fetal bovine serum and (3-Aminopropyl)triethoxysilane (APTES) 10% v/v were acquired from Thermofisher (Massachusetts, USA). Petri dishes and all laboratory equipment were obtained from Merck (Darmstadt, DE).

### Cell preparation

SH-SY5Y Neuroblastoma (NB) cell lines (CRL-2266) derived from metastatic bone tumors, obtained from the cell repository from the OPBG were kindly provided by Dr. Di Giannatale. These cells are known to differentiate in N-type (neuronal) and S-type (substrate-adherent) and have the additional characteristic of being capable of growing in adhesion or in suspension, making them the ideal candidates for our experiments (Kovalevich and Langford, 2013; Bell et al., 2013).

Cells were cultured in DMEM low glucose supplemented with 10% Fetal bovine serum, 1% penicillin-streptomycin and 1% L-Glutamine. Cells were incubated at 37°C in a humidified atmosphere with 5**%** CO_2_. Cells were seeded 24–48 h prior to measurements on plastic Petri dishes. Medium composition, cell culture density and temperature were kept constant throughout all experiments.

### Setup description

For all our experiments we have used two interchangeable setups, based on two atomic force microscopes (AFM): a Park NX-12 (Park Systems, Suwon, Korea) and a Nanosurf Flex (Nanosurf AG, Liestal, Switzerland). These microscopes were mounted on an Olympus IX-9 inverted optical microscope (Olympus Corporation, Tokio, Japan) equipped with a high-resolution Progres MFCool digital camera (Jenoptik, Germany) and an active antivibration table, to ensure that environmental noise did not influence the measurements. This setup allowed performing concurrently all the measurements on the chosen cells. To ensure the measured effects were only correlated to cellular behavior and not due to external factors, all experiments were carried out in a controlled environment, kept at 37°C in 5% CO_2_ and in a fully humidified environment throughout the entire measurement run.

The optical microscopy images were used to monitor the behavior of the cells both on the sensor and on the Petri dish and were collected every 20 s using a 40x objective. By using a semi-automated cell tracking system [Fiji, a distribution of the freeware ImageJ (Schindelin et al., 2012)], we followed the movements of these P-cells, highlighting the path followed by the cells after every image.

Regarding the nanomotion setup, we chose commercial AFM cantilevers as sensors, namely Bruker ONP-10 tipless AFM cantilevers (Bruker Corporation, Massachusetts, USA), choosing the sensor with a nominal elastic constant of 0.12 N/m. Prior to all experiments, the sensors were calibrated using the built-thermal-noise routines to determine the resonant frequency and the corresponding mechanical properties of the sensor (Hutter and Bechhoefer, 1993). The nanomotion signal was acquired using custom LabView software to control a NI USB-4431 card (National Instruments, USA) collecting the nanoscale oscillations of the sensor caused by the oscillations of the cells at a 15 kHz rate. We analyzed this data using a custom Labview software, to calculate the variance of the nanomotion signal over small time-chunks (typically 10–60 s) (Venturelli et al., 2020).

### Sensor preparation and cell immobilization

Figure 1 shows a sketch view of the setup and, in the inset, the field of view in a typical experiment is shown, with small and large clusters of P-cells passing underneath the sensor.

The setup used to monitor and detect vibrations of neuronal cells is similar to a conventional nanomotion setup, as described in detail in previous works (Longo et al., 2013; Venturelli et al., 2020). At first, the sensors were washed in ultrapure water, functionalized by 10 min exposure to APTES 10% v/v which was followed by thorough rinsing in ultrapure water and immediate transfer to the AFM for immediate use.

Next, we placed growing medium and living NB cells in a Petri dish which was not functionalized. This substrate allows a weak cellular attachment but does not stimulate complete cellular adhesion, thus placing the cells in an environment in which their innate tendency to grow in adhesion is impeded, possibly stimulating environmental sensing and interaction. The sensor was then brought in the near vicinity to the surface by using the AFM's motors, and single cells or small clusters were identified for collection. To do this, we pressed the sensor against chosen specimens allowing the functionalization of the sensor to stimulate the cell's adhesion. After 1 min of pressure (maximum applied pressure 20 nN), we retracted the sensor to a distance from the Petri dish surface of 100 or 200 μm. During this whole procedure, we used the optical microscope first to determine which cells to attach and next to monitor the firm cell adhesion to the sensor. The optical microscope was also used to monitor over time both the sensor bearing the NB cells (which we call S-cells) and the other NB cells present on the Petri dish (the P-cells). Each experiment lasted at least 4 h (with some measurements rounding up to up to 7 h) and was divided into 30- or 40-min chunks for the analysis.

In the typical experiment the motion of the cells, both the P-cells and the S-cells, were combined with the analysis of the time-dependent fluctuations induced by the S-cells on the sensor. The variance of the nanomotion signal was directly related to the activity of the S-cells in the different environmental conditions (Kohler et al., 2019; Girasole et al., 2023), while the movements of the P-cells gave us an insight on the interaction between the NB cells.

Notably, even in a small receptacle such as a Petri dish, the medium underneath the sensor can exhibit a flux, which drives the movements of the P-cells. We were able to identify this flux in terms of speed and direction by following the small particulate in the growing medium. We focused on the alteration of these movements correlated to these medium microcurrents when influenced by the presence of the sensor and of the S-cells, mediated by the oscillations of the S-cells (as depicted in Figure 1).

### Cell health estimates

Optical images evidenced that the cells exhibited normal behavior, including formation of filopodia and substrate probing, which were determined to be signals of good viability.

As additional control, other NB cells were kept in the same environmental conditions side-by-side with the cells under investigation, and the viability and wellbeing of these control cells were verified at the end of each experiment.

### Statistical analysis

The presented results were replicated in more than 30 independent experiments from distinct preparations, and several different interaction events were collected throughout each experiment. Bearing this in mind, there is a point to be highlighted regarding the variability of each experimental run. The number, position and activity of the P-cells as well as of the S-cells is difficult to control and to categorize. Indeed, even if we can control the position and number of S-cells at the beginning of each experiment, they were free to move, even if on a very small platform, thus we had no control over their displacement during the experiment. Furthermore, we had no control over when and where the P-cells appear and at what distance they will pass in the vicinity of the S-cells. This means that a completely quantitative determination of the cell-cell interaction, a priori, is impossible. The only statistical determination we can provide is a statistical analysis of the average cell-cell distance at which we can determine that an interaction is underway, through which we have estimated the size of the approximate interaction-sphere within which the relative effect can be observed.

## Results

### Large-scale movements of NB cells

In all the experiments the optical images evidenced how both the cells attached to the sensor (S-cells) and those moving on the surface of the Petri dish (P-cells) shifted and moved, often through a roto-translational pattern. The available space for their motion was very different: while the S-cells were observed vibrating and moving over the nano sensor, the P-cells moved on much larger distances, often entering and exiting the field of view of the optical microscope.

**Figure 2:**
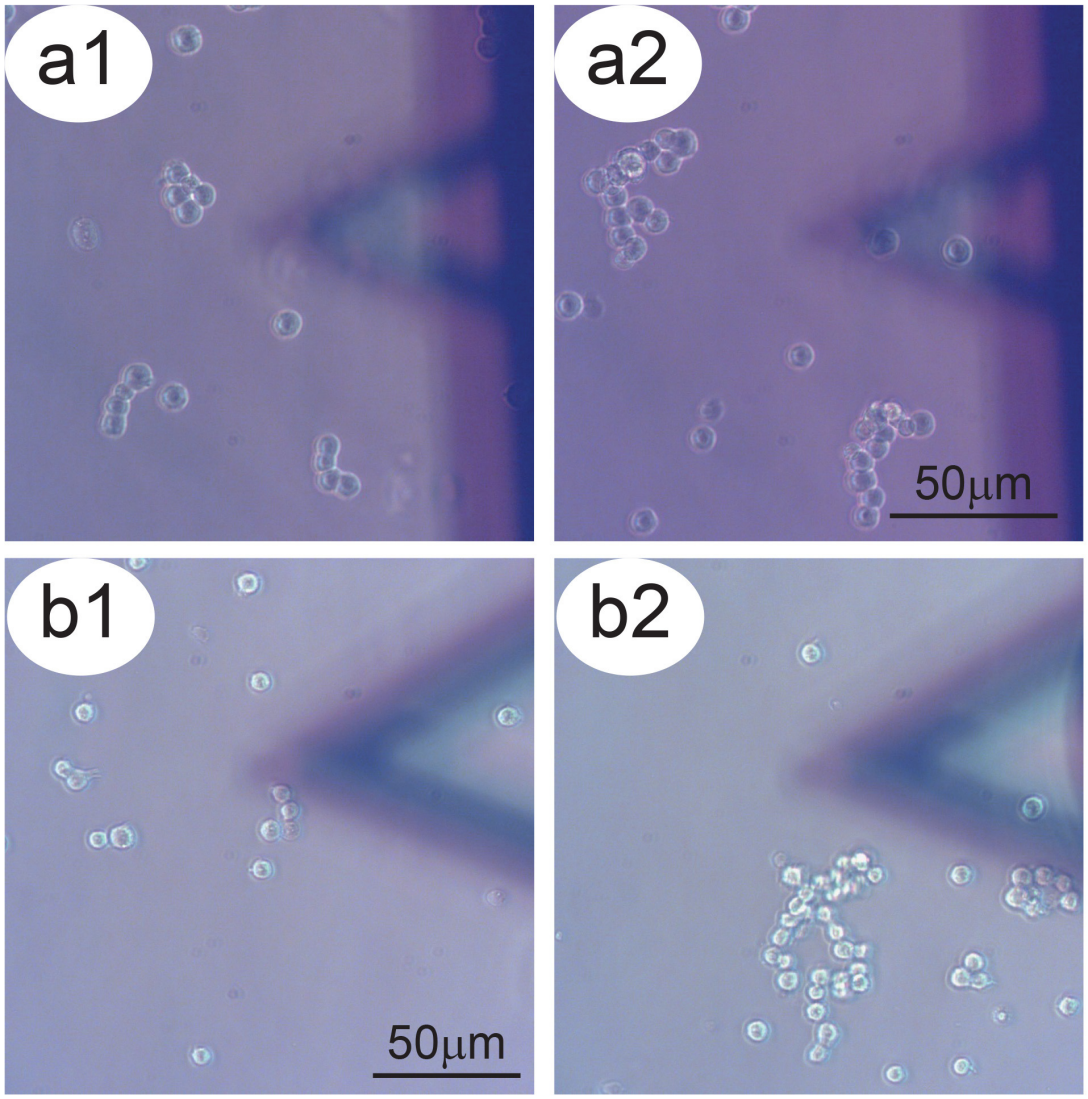
Aggregation tendency of P-cells. Panels a1–a2 and b1–b2: Two examples of NB cells which tend, over time, to aggregate to form larger clusters. In the foreground, the sensor bearing S-cells.

The first and most common behavior of the P-cells was that they moved freely over the Petri dish substrate to find and make contact with other cells (Figure 2). In this way, they typically formed larger clusters. This appears to constitute a major driving force influencing the activity of freely moving NB cells which should be considered in the interpretation of the data. A second effect influencing the movements of P-cells was the micro-dynamics of the growth medium. Small differences in liquid pressure in the Petri dish produced local fluid currents, which can be seen through the drifting of small particulates in the medium. This flow directed the movement of the P-cells, driving them in specific directions. We highlighted such trajectories which were derived from the time-lapse videos of the P-cells in the presence of a sensor. The resulting overlay lines and the corresponding displacement graphs depict a typical scenario in which clusters of P-cells pass in the vicinity and underneath the sensor and allow comparing the behavior of the cells with cases in which the cells pass far from the sensor and from the cells placed on it.

When no S-cells were present (Supplementary Figure 1a), the cells were driven by the microflow of the medium and passed in view with approximately linear paths, unaltered by the presence of the bare sensor (Supplementary Figures 1b–d).

**Figure 3:**
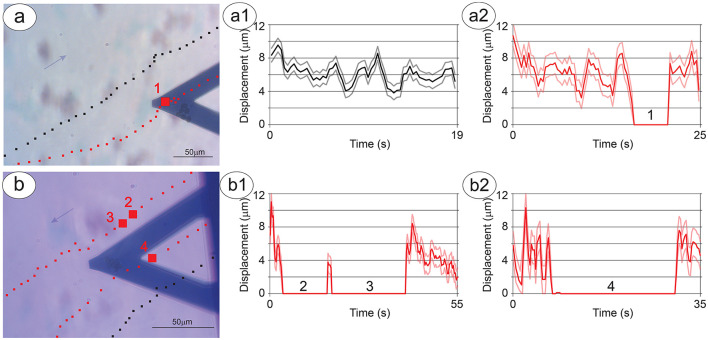
Dynamics of the interaction between P-cells and S-cells. The trajectories (series of black or red squares on the optical image) and displacement (corresponding a1–a2 and b1–b2 graphs) of a cell cluster over time. **(A)** Shows a cluster stopping near the S-cells (red squares with a larger square corresponding to the area of cell-cell interaction) and a nearby cluster which does not change its trajectory (black squares). **(B)** Shows two clusters stopping near the S-cells (red squares with larger squares and numbers corresponding to the areas of cell-cell interaction) and a nearby cluster which passes under the sensor but does not change its trajectory (black squares). The scale bar indicates 50 mm and the lighter variance curves indicate the error in the displacement measurements.

When NB cells were present on the sensor, while P-cells passing far from the S-cells appeared to continue an unaltered path (Figures 3a, a1), the P-cell clusters that passed in the close vicinity and underneath the S-cells experienced a modification of their motion, such as slowing down or brief stops (Figure 3, a2), up to a transient or permanent stop when in close proximity to the sensor (Figures 3b, b1, b2), even against the micro-currents of the growth-medium (see the blue arrows in Figures 3a, b). Some cases exhibit a large deflection of the cell path or a combination of different P-cell clusters to interact with the NB cells on the sensor (Supplementary Figure 2, Supplementary Movie 1).

Remarkably, in most cases, the interaction between the P-cells and the S-cells starts before the former cells pass near the sensor, and in presence of a medium flux, with the P-cells still upwind to the S-cells (Figure 3b, Supplementary Figure 2). Interestingly, there are cases in which the P-cells move against the medium flow, reducing their velocity and even deviating their trajectory to approach the specimens on the sensor. In addition, the time needed for the clusters of P-cells to alter their motion is fast, with changes in speed and direction happening in less than the time between two subsequent optical images (i.e., 20 s).

The observed cell-cell interactions evidence additional peculiar behaviors. In a remarkable experiment, clusters of P-cells have partially detached from the substrate, moving toward direct contact with the cells on the sensor. This is particularly interesting as these S-cells were suspended over the Petri dish surface at 100 microns on the vertical axis (Supplementary Figure 3, Supplementary Movie 2).

These dynamics suggest that the mechanisms underlying the cell-cell interaction point toward the formation of large cell aggregates and are strong enough to produce substantial and unexpected consequences on the cell behavior.

**Figure 4:**
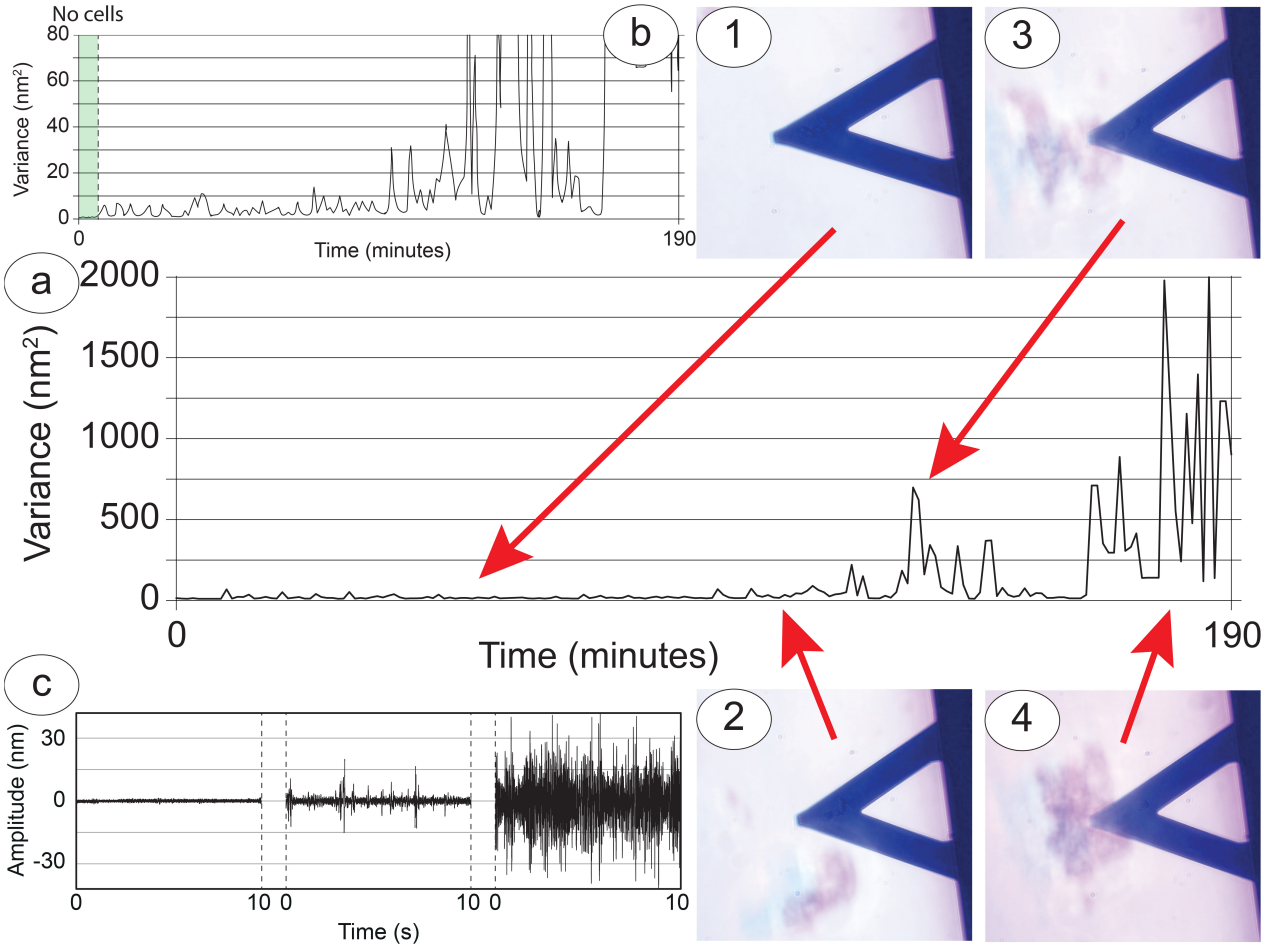
Cellular nanoscale vibration response of the S-cells to the nearby presence of the P-cells. **(A)** Nanomotion variance during an entire experiment, lasting more than 3 h. **(B)** Zoom in to highlight the behavior at lower variance values. Panels 1 to 4: Snapshots of interesting cell-cell interactions: the nanomotion variance increases according to the size and distance of the cluster of P-cells which have approached the sensor. **(C)** Typical amplitudes collected from the sensor in correspondence to zones 1 and 2 (right curve), zone 3 (center curve) and zone 4 (right curve).

Regarding the S-cells, these are limited in their movements by the geometry of our setup but at the same time tend to interact with the P-cells by shifting and moving toward them when they come by (Figure 4, panels 1–4). A very interesting characteristic of these cells is that their behavior depends greatly on their number. In experiments when only one or two cells were loaded on the sensor, they appeared to move on the sensor, exploring the surrounding environment, possibly focused on the search for other cells. This movement pattern of single cells often brought them to detach from the sensor (Supplementary Figure 4), especially when some cluster of P-cells passed in the vicinity, thus showing a preference toward the cell-to-cell contact instead of the functionalized surface of the sensor.

Because of the numerous possible configurations of medium flow, the varying number and positions of S-cells, and the diverse abundance and clustering patterns of P-cells, a full statistical analysis of how sensor and P-cells interact is unfeasible. Therefore, we measured the distance between the cells when a change in their movement, indicating an interaction, could be detected (Supplementary Figure 5a). We used these values to define the range of interaction between the P-cells and the S-cells (Supplementary information 2).

### Nanomotion

In experimental conditions where cell-cell interactions occur, the movements recorded by the nanosensor provide an insight into the behavior and status of the S-cells and during their interaction with the P-cells. A typical nanomotion signal of an experiment can be used to compare variance (Figures 4a, b) and amplitude of the oscillations (Figure 4c) with the optical images (Figure 4, time-points 1–4). Through this comparison we can divide the experiment into different sections. At first there are five well-attached cells onto the sensor, resulting in an overall movement transferred to the sensor which has a low amplitude and is constant over time (Figure 4, time-point 1). When a large cluster of P-cells approaches, this excites at least two S-cells which start to shift on the sensor in a roto-translation pattern, causing a slight but measurable increase in the overall fluctuations of the sensor, with peculiar spikes in the detected signals (Figure 4, time-point 2). Even after the departure of the cluster, the cells on the sensor maintain their increased roto-translational activity.

When a second larger cluster arrives near the sensor and interacts with the S-cells, these increase their activity, moving on the sensor and extending filopodia or neurites, and this produces a significant increase of the nanomotion signal (Figure 4, time-point 3). The nanomotion pattern is diverse, with large spikes and an overall large amplitude of the fluctuations. Finally, when a very large cluster of P-cells approaches and stays under the sensor, interacting with the S-cells, the cluster of cells splits, and the motile activity of each cell increases. This causes a further increase in the nanomotion signal with much higher oscillations and eventually resulting in some of the S-cells, at that point no more bound to the sensor cluster, detaching from the sensor to join the larger cluster on the Petri dish (Figure 4, time-point 4).

The alterations in the behavior of S-cells when P-cells are approaching has been consistently observed across multiple experiments (*n* = 5), suggesting a reproducible and generalizable cellular response.

## Discussion

We presented a series of experiments designed to highlight and characterize, at micro and nanoscale and in a controlled geometry, the interactions between cells. The goal was to estimate the possible role for acoustic fields in the cell-cell interaction process even at the single cell level.

We focused on a simplified nanoscale system consisting of a small cluster of NB cells geometrically constrained to a small flat surface, interacting with a larger number of other NB cells freely moving on a Petri dish which did not favor their adhesion. This condition stimulated in P-cells the need to explore the environment, searching for a surface where to adhere or for other cells to form larger self-sustained clusters and in the S-cells the tendency to communicate with other cells and an amplified activity which was measured by the nanomotion sensor.

Our experiments showed that the activity of the cells is dominated by a general trend leading to the formation of large clusters of NB aggregates. This general behavior must be mediated by forces acting at the cellular scale and is expected to be limited and modulated by biological, physical and environmental factors.

The experimental setup that we propose presents a two-fold advantage. On one side it provides a unique environment to stimulate and observe the cellular interactions. On the other side, the nanomotion sensor has the capability to monitor the cells' activity in real time and to quantify their activity during their homeostasis or during cell-cell interactions.

Indeed, the results shown in Figure 4 point toward a large increase in activity of the S-cells during interaction with P-cell clusters, an interaction that appears to be mediated by relevant cellular communication. In fact, many of our observations have evidenced how, in absence of external measurable forces, P-cells have altered in a large manner their motion in the vicinity of the S-cells, even detaching from the substrate (Supplementary Figure 3). In several cases, such alterations of the free motion of the P-cells occurred against the flow of the medium, that is, against the environmental force gradient (Figure 3, Supplementary Figure 2).

While a purely chemical interaction is commonly considered to be the main actor in cell-cell communication, the upwind directional responses observed in our experiments cannot be simply explained through simple chemical communication. Such a fast, upwind and complex geometry of interaction suggests that the cells could exploit their strong tendency to communicate by tapping into different kinds of communication mechanisms, including those driven by mechanical stimulation.

The data shown in Supplementary Figure 5a indicates that the cell-cell interactions depend on many parameters such as the number of cells, their status and the strength of the medium flow. In any case, we were never able to identify interactions which exceeded 300 microns in distance between clusters. To understand if the S-cells' oscillations were sufficient to produce a mechanical wave that could be detected by the P-cells, we performed a semi-quantitative evaluation of our data (Supplementary information 2). Considering our geometry and the characteristics of the cells and of the medium, we were able to determine that the oscillations generated by the S-cells and integrated by the sensor in a clear and coherent signal, can produce an oscillating mechanical field which has a sufficient amplitude to interact on cells distant even several hundreds of microns. According to the measured value of sensor oscillation, this traveling field has the strength to determine membrane deformation on the target P-cells which, in our experimental condition, can be predicted to activate the mechano-transduction mediated by PIEZO proteins and by integrins (Kumar and Weaver, 2009; Lin et al., 2019; Baratchi et al., 2024; Niu et al., 2022; Wang and Ha, 2013; Jo et al., 2022). Furthermore, our interpretative model includes the viscous behavior of the culture medium (a real fluid) which, through the energy draining occurring in the Stokes layer (Sader, 1998), allows understanding why the effects on the P-cells were observed only within few hundreds of micron from the source.

In fact, the calculated range of such Stokes layer in our experimental conditions, agrees with the maximum distance at which we unambiguously identified cell-cell interactions (Supplementary Figure 5b). It is worth noting that, on a larger scale of biological aggregation, signaling through these kinds of oscillations are associated with acoustic waves, and we can suggest that these can have an impact also on the collective activity of even the smallest building blocks of living organisms (i.e., cell clusters).

Overall, mechanical oscillations produced by cellular vibrations in a fluid environment can generate a distortion of the density/pressure field that can be detected and transduced by target cells through mechano-sensing proteins and result in cellular response. We have presented evidence that suggests that such acoustic waves can be scaled down even to single cell interactions.

While a direct measurement of the acoustic waves is impossible in our setup (acoustic waves in liquids are usually measured by hydrophones, which are bulky and do not have the sensibility to measure waves at very short distances), our model of cell-cell interaction supports this conclusion.

Obviously, the complexity of a real experiment cannot be completely reflected in this simplified model. Indeed, extensive statistical analysis is complex, since our experimental setup welcomes biological variability, heterogeneity in cellular response and the randomness of a real-life scenario to better understand the collective behavior of the NB cells. Furthermore, our model doesn't consider effects associated with multiple reflections of the acoustic fields or the geometrical limitations of the cell-wave interaction, which could modulate the effectiveness of the biological transduction.

Further confirmation of the proposed role for acoustic-based communication would come from dedicated experiments involving fluorescent tags on mechano-transductive proteins to better highlight the chemical signaling pathways and their alterations in presence of acoustic waves. Similarly, investigating the effect of specific protein inhibitors or that of drugs known to alter cell-cell interactions would provide a better biological characterization for this new interaction mechanism.

Within the limits of our setup and model, we propose that an acoustic field can be invoked to justify, directly or indirectly, the counter-intuitive cellular behavior observed in our experiments, especially considering that acoustic fields may act through multiple mechanisms. For instance, the induction of mechanical waves in the liquid may contribute to a greater diffusion of neurotransmitters in the culture medium or it may increase the availability of the signal molecules dispersed in the buffer, contributing to an acoustic enhancement of the “conventional” chemical signaling of the cells.

## Data Availability

The raw data supporting the conclusions of this article will be made available by the authors, without undue reservation.
