# Supplementary material for: Toward a role for the acoustic field in cells interaction
Source: Front Syst Neurosci. 2025 Jun 18;19:1484769. doi: 10.3389/fnsys.2025.1484769 (PMC12213812; doi:10.3389/fnsys.2025.1484769)
Supplement: Supplementary file 3 [file Data_Sheet_1.docx]

**Toward a role for the acoustic field in cells interaction**

*M. Girasole1, P.F. Moretti2*, A. Di Giannatale3, V. Di Paolo3, A. Galardi3, S. Lampis3, S. Dinarelli1, G. Longo1**

*1 Institute of the Structure of Matter, Consiglio Nazionale delle Ricerche, ISM-CNR, Rome, Italy*

*2 Department of Earth System Sciences and Environmental Technologies, Consiglio Nazionale delle Ricerche, Rome, Italy*

*3 Hematology/Oncology and Cell and Gene Therapy Unit, Bambino Gesù Children’s Hospital, Istituto di Ricovero e Cura a Carattere Scientifico - IRCCS, Rome, Italy.*

**Corresponding authors: longo@ism.cnr.it, pierfrancesco.moretti@cnr.it*

**Supplementary information 1: Control experiment: P-cells with no S-cells**

We performed a series of experiments to evaluate if the mere presence of the sensor was enough to elicit an alteration of the path of the P-cells. To do this we repeated the experiments using the very same setup but without the collection of sensor cells. Indeed, in this configuration, we have a bare sensor with P-cells moving underneath it.

As seen in Figure S1, there are several single cells and cell clusters moving on the surface of the Petri dish and directly underneath the sensor, yet none shows any kind of alteration in their trail due to the presence of the structure.


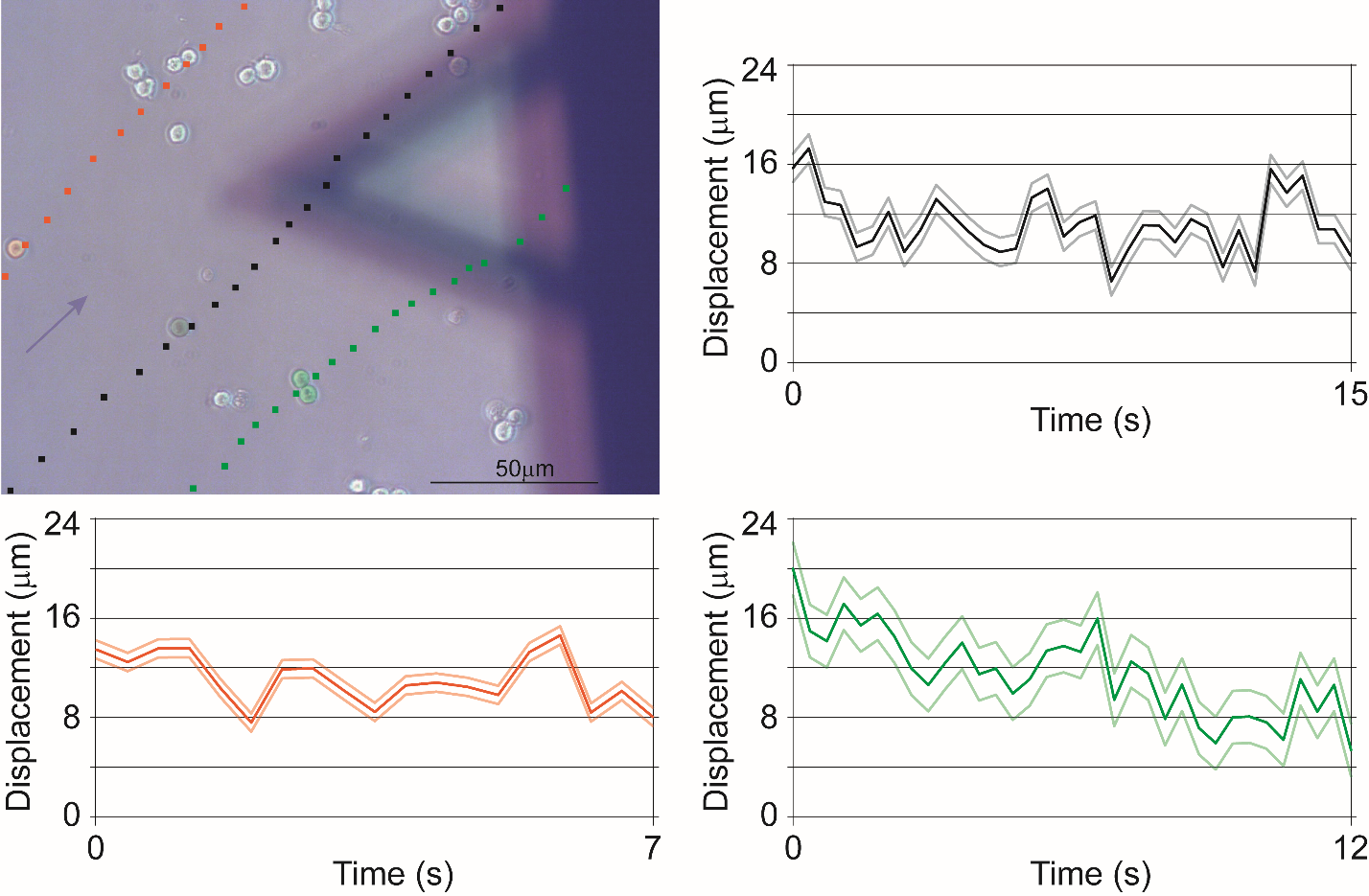


**Supplementary Figure 1**: Control with no sensor cells. Panel a: the setup with the path of three cells or cell clusters highlighted in red, black and green. The corresponding specimens are evidenced in the corresponding colour. The blue arrow indicates the medium flux driving the cell movement. Panels b, c and d show the step-by-step displacement of the three specimens, highlighting how none of them reduce their movement before, during or after their passage under the sensor.

**Additional supplementary figures:**


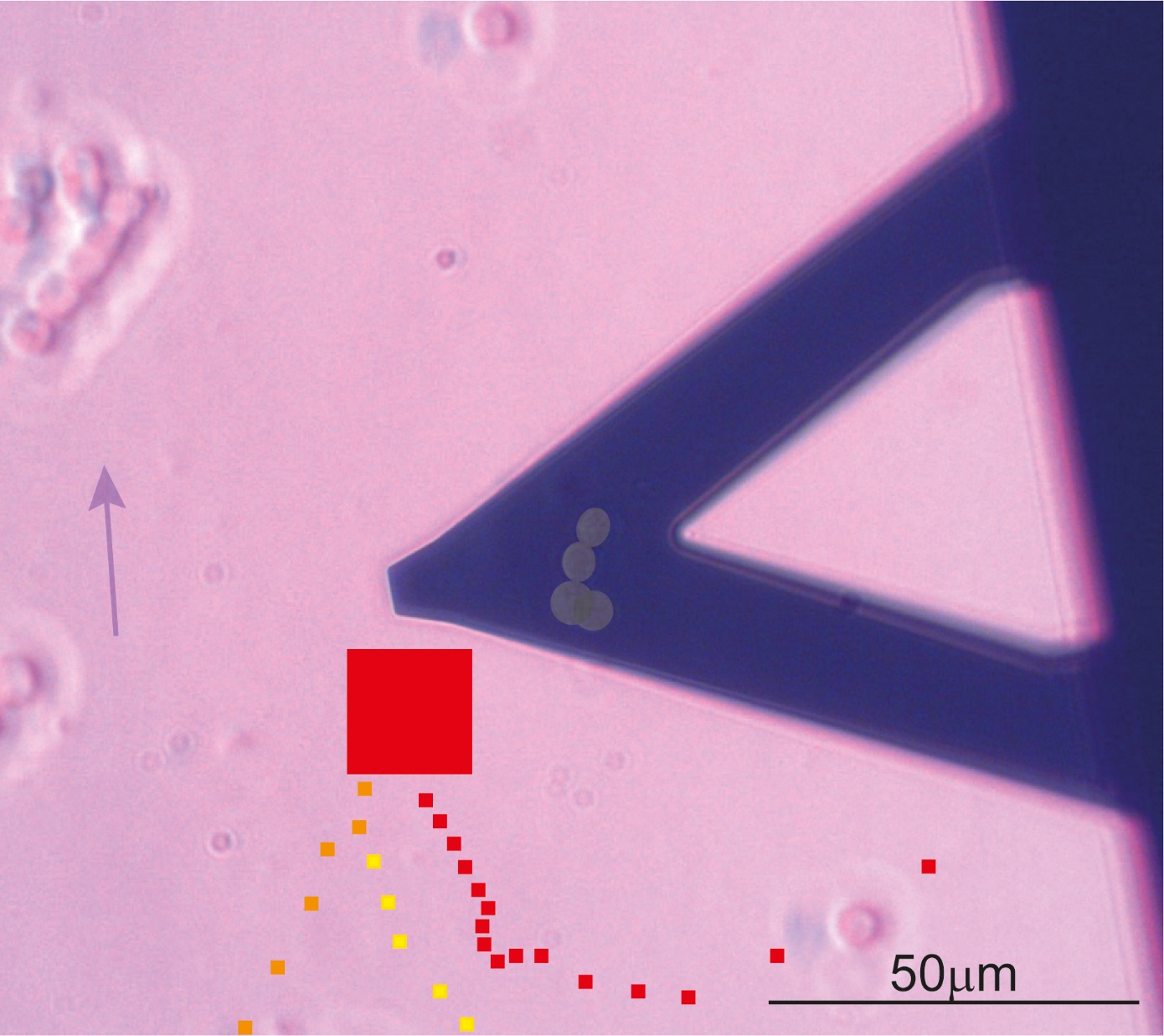


**Supplementary Figure 2:** Dynamics of the interaction between P-cells and sensor cells: a P-cell cluster (red dots) interacts with a single cell (yellow dots) and a cell cluster (orange dots) to approach the S-cells and form a larger NB cluster. This lingers in the immediate vicinity of the sensor, in an overwind position, to interact with the S-cells. The full dynamics are shown in Supplementary Movie 1.


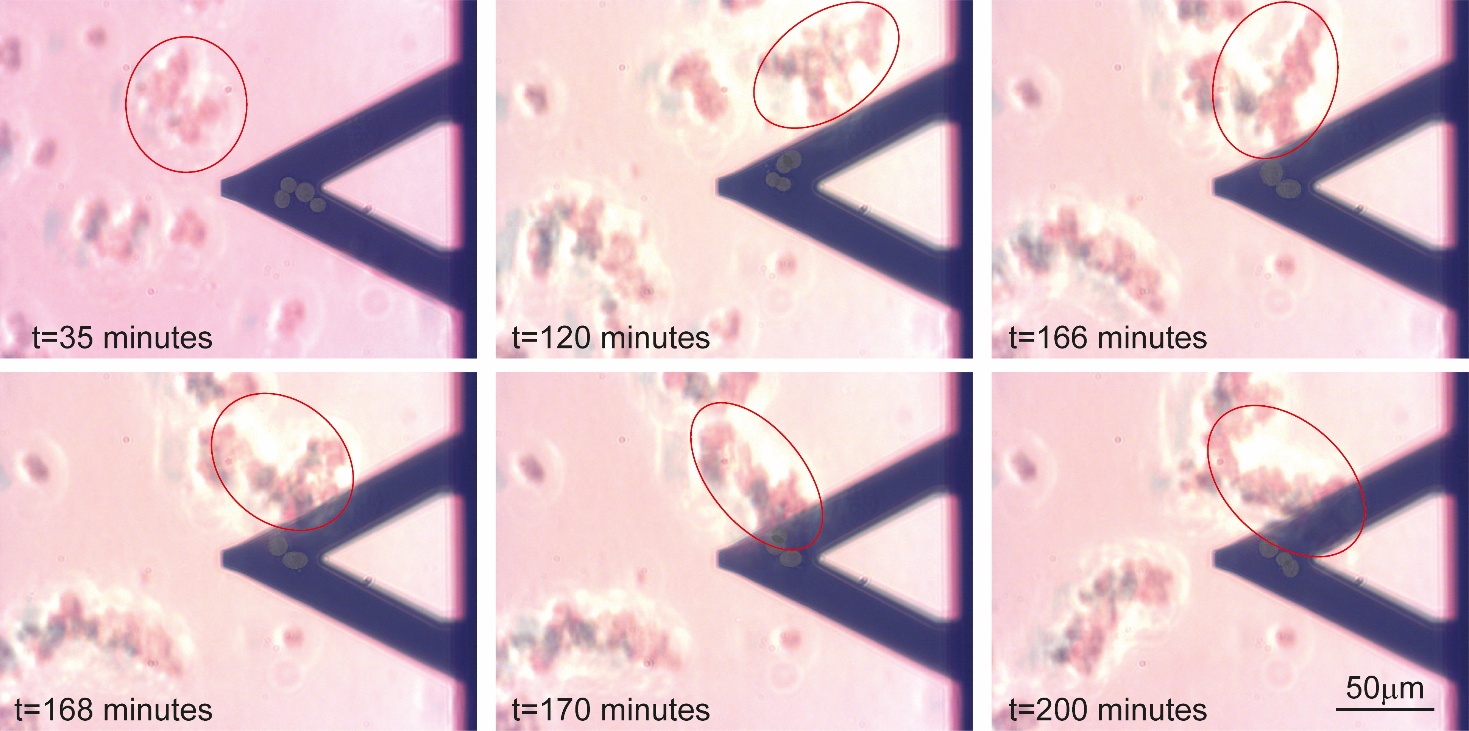


**Supplementary Figure 3**: Peculiar behaviour of a P-cell cluster approaching S-cells. A cluster (red oval) approaches the sensor and (at 170 to 200 minutes timepoints) detaches from the Petri surface to make contact with the S-cells. The three-dimensional movement is also shown in the corresponding Supplementary Movie 2.


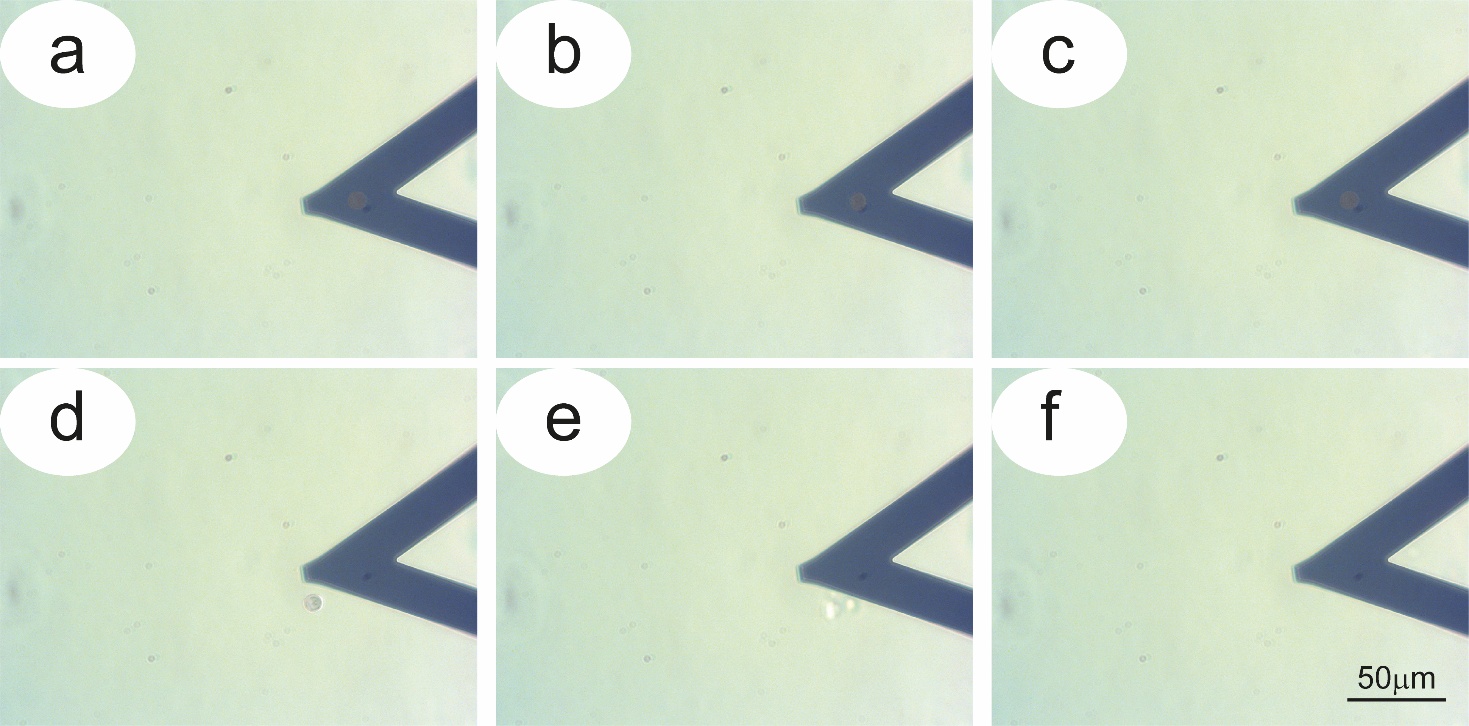


**Supplementary Figure 4**: Images depicting the typical behaviour of single NBs when attached to the sensor. The cell probes the sensor surface to detect other cells (Panels a, b and c) and in Panel d detaches disappearing in the background (Panels e and f).

**Supplementary information 2: A semi-quantitative evaluation of the cell-cell interaction**

Our results evidence how we were able to identify biological interactions between NBs separated even by several hundreds of microns. Often, such interactions were shown to happen in conditions that do not support simple schemes driven by chemical or even electromagnetic interactions. Thus, we want to explore the possibility that cellular oscillations at the nanoscale (which on a larger scale could be identified as acoustic waves) can be sufficient to stimulate such cell-cell interactions.

To determine if the cells could, through their movements, stimulate such effects, we have investigated 4 major points:

1) The generation of a mechanical oscillating field i.e. an acoustic field

2) The transmission of the acoustic field from the source (the sensor cells) to the destination (the P-cells)

3) The ability of the P-cells to detect this signal

4) Re-evaluation of the model

**S2.1 The generation of the field**

The cells under investigation are positioned on a sensor that transduces the cellular vibrations into measurable oscillations. The oscillations of the sensor measure the cellular motility and allow studying their characteristics under varying environmental conditions. Furthermore, these cellular oscillations, transduced by the sensor, also produce a vibrational field that propagates following the same laws of an acoustic wave in the liquid. The sensor does not amplify the cellular oscillations, but rather its effect is to integrate the individual cellular motilities into an overall vibration. The resulting acoustic wave in the medium can be associated with a clean and coherent signal, although complex in its time evolution. Such waves will perturb the surrounding space in which, at the same time, other cells are generating smaller individual vibrational fields.

**S2.2 The transmission of the signal**

Acoustic waves in liquids are usually measured by hydrophones, which are bulky, have limited sensitivity and are not calibrated to determine the amplitude of the oscillations at distances of few hundreds of microns. Despite this, the classic fluid-dynamics theory that regulates acoustic waves can be used to evaluate the energy transmission associated with the propagation of a wave in a fluid.

The factors that contribute to damping are essentially (i) the absorption of the medium (including the presence of solutes, macromolecules and, most important, viscoelastic elements such as other cells in suspension) and (ii) the geometric divergence (arising from the conservation of energy).[1]

Here, it is important to note that any estimate of the attenuation of an acoustic field leads to the conclusion that, even in our experimental conditions, the field can travel for lengths enormously greater than the spatial scale of our experiment (e.g. in seawater the signal can travel even for kilometres at frequencies below 1 kHz and even much more at lower frequencies).[2] Therefore, the transmission of the signal in liquid (even considering DMEM instead of water) does not pose a challenge for the transmission of the wave.

**S2.3: The ability of the P-cells to detect the waves**

Once generated by the S-cells, the acoustic wave will certainly reach the P-cells, which are viscoelastic structures biologically prone to interact with the environment. Mechano-sensing and mechano-transduction constitute a fundamental pattern of cellular response to mechanical stimuli that is present in various cellular systems (e.g. neurons, RBCs, cardiomyocytes and others).[3; 4; 5; 6; 7] These patterns are attracting growing interest in the scientific community following the accumulation of evidence on their role in a variety of biological phenomena, including the three-dimensional organization of organoids, cell migration and the development of pathologies (e.g. metastasis of cancer cells). In the case of neurons, mechano-transduction is of fundamental importance for the perception of sensory stimuli, for the development of the central nervous system and a variety of other phenomena.[8]

From the molecular point of view, mechano-transduction in neurons relies on the role of several proteins, among which PIEZO 1,2 and integrins stand out. PIEZO proteins, known to be low-threshold, high-sensitivity mechanical sensors[9], are membrane channels for mono- and bivalent cations including Na^+^; K^*^ and Ca^++^ and have been shown to remotely regulate the activity of other channels[10] such as TREK, which are associated with potassium currents. Integrins, on the other hand, are mechanosensitive proteins that connect the extracellular matrix (ECM) with the fibers of the cellular cytoskeleton and can activate a biochemical signalling cascade following a conformational transition associated with an external mechanical stimulus. It is precisely this mechano-transductive role that ensures that integrins remain expressed even in suspended neurons (e.g. when treated with trypsin), that are very similar to those studied in our experiments.

For both these proteins, the mechanical properties that mediate the dynamics of the response to external stimuli have been studied.[9; 11; 12] In particular, for PIEZO 1, an elastic constant (K_el_) of 7 pN/nm was measured through HS-AFM [13] and, despite differences due to the membrane composition and the modalities of application of the stimulus, it has been shown that forces in the range 20-40pN are sufficient to activate the mechano-transduction. Integrins are also able to respond to force stimuli in the same range, although the molecular mechanism that mediates their activation is different and a greater sensitivity to external stimuli has been proposed for these proteins.[12; 14]

In our experimental case, it is possible to perform an estimate of the forces transmitted to the cells due to the oscillation of the sensor induced by the movement of the S-cells. Indeed, in harmonic approximation (F=K_el_*Δx) and assuming, as stated previously, a K_el_ of the membrane in the order of 7 pN/nm, the application of an oscillating acoustic field capable of inducing a deformation around 3-6nm would guarantee the activation of the protein response. On the other hand, our experimental data highlights that the amplitudes of the isolated S-cell oscillations (transduced and integrated by the lever) are typically on the scale of 2-10 nm. (see for instance Fig. 3 panel c). Furthermore, single cells determine smaller sensor oscillation (1-3 nm), while larger clusters can produce larger, and likely, more effective signals. Remarkably, when S-cells come in the vicinity of P-cells, their resulting enhanced motility produces vibrations whose amplitude can reach several tens of nm.

Thus, the cell vibration-associated signals observed in our experiments have magnitudes compatible with activation of mechano-transductive pathways on the P-cells

**S2.4 A re-evaluation of the distance-dependence data.**

Our experimental runs have shown how the interaction between S-cells and P-cells does not occur at all cell-cell distance, and we did not observe dynamic, unequivocal alterations of the motion of P-cells at distances greater than, 250-300 μm from the S-cells. (Supplementary Figure 5 Panel a) Furthermore, the number of observed “anomalies” (such as unexpected change in speed and direction, cell slowing, cell-cell contact etc.) decreases as the distance increases (even maintaining the same geometry of the system).

According to the classical theory, the behaviour of a real fluid in the presence of an oscillator is described through the thickness of a Stokes layer (also called oscillatory boundary layer thickness). In an intuitive view, the Stokes layer indicates the region of space in which the motion of the fluid, dominated by the oscillatory effects of the sensor and in the presence of viscous damping, is more relevant and evidently more perturbative for a hypothetically exposed biosystem.[15; 16] Outside of this layer, the effects of the movements in the fluid are mainly caused by pseudo-elastic deformation of the fluid which, as previously stated, has very little attenuation.

The reference formula for the Stokes layer depth is:

$$\delta=\sqrt{\frac{\eta}{\pi\rho f}}$$

The Stokes layer thickness (δ) gives the scale dependence of an exponential decay for the amplitude and depends on the frequency (f) of the acoustic field and on liquid properties such as viscosity (η) and density (ρ). Numerical calculations for relevant experimental conditions reveal that in our cases, if the interaction is mediated by a cell-generated oscillating wave, the effect takes place almost entirely in the Stokes layer. This is particularly true for the lowest frequencies, which correspond to larger Stokes layer thickness (e.g. 110 μm at 20 Hz; 330 μm at 2Hz; Supplementary Figure 5 Panel b).

Overall, the presence and thickness of the Stokes layer, where most of the field energy is dissipated and the liquid motion follows the sensor oscillation, is relevant to explain the fluid dynamics of our experiment and the Stokes layer seems to represent the perfect limiting factor for the observed cell-cell interactions. This agrees with the intuitive idea that the biological effects (e.g. mechano-transduction) require significant liquid dynamics and larger energy fields. It is also suggestive that the most effective frequencies of the field reflect time scales where many biological processes actually take place.


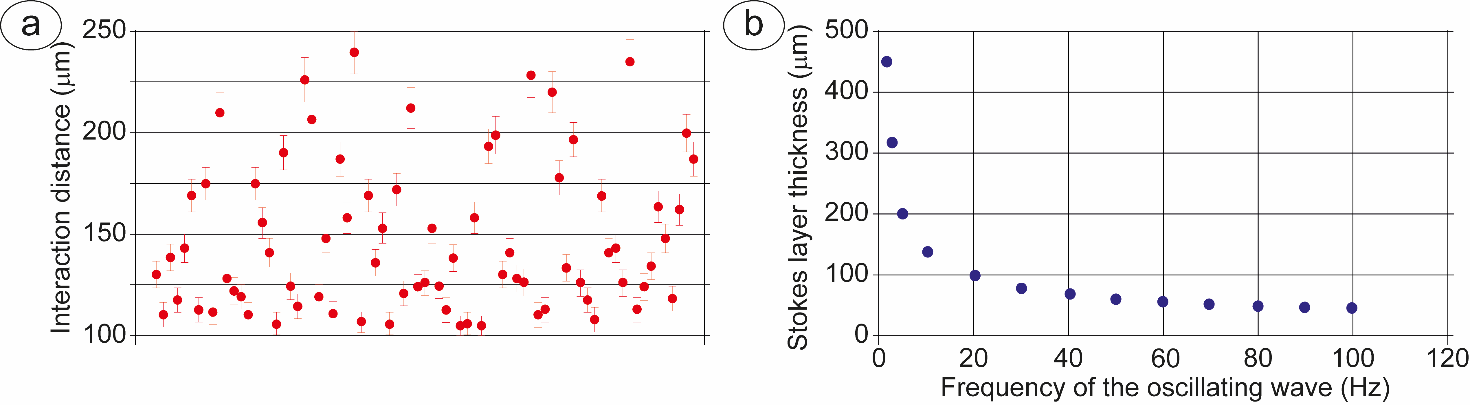


**Supplementary Figure 5**: Panel a: Scatter plot of the distances at which the interactions between P-cells and S-cells can be determined. Panel b: Thickness of the Stokes layer as function of the frequency

It is worth noting that this interpretative model has simplified some aspects of the interactions. For example, we have not taken into account possible geometrical effects caused by the field-cell interaction, that can modulate the membrane deformation and consequently the efficiency of mechano-transduction.

Moreover, (finite) boundary conditions typical of this experimental setup have not been considered: multiple reflections from the bottom or from the walls of the Petri dish can occur and can modulate the effective distribution of the fields. All these factors, obviously, can potentially play a role for the effectiveness of the other proposed mechanisms (e.g. dispersion of neurotransmitters and modulation of their bio-availability).

**Additional references**

[1] M.A. Ainslie, and J.G. McColm, A simplified formula for viscous and chemical absorption in sea water. The Journal of the Acoustical Society of America 103 (1998) 1671-1672.

[2] G. Grelowska, and E. Kozaczka, Underwater Acoustic Imaging of the Sea. Archives of Acoustics 39 (2014) 439-452.

[3] S. Dinarelli, M. Girasole, P. Spitalieri, R.V. Talarico, M. Murdocca, A. Botta, G. Novelli, R. Mango, F. Sangiuolo, and G. Longo, AFM nano-mechanical study of the beating profile of hiPSC-derived cardiomyocytes beating bodies WT and DM1. Journal of Molecular Recognition 0 (2018) e2725.

[4] S. Dinarelli, G. Longo, A. Francioso, L. Mosca, and M. Girasole, Mechano-Transduction Boosts the Aging Effects in Human Erythrocytes Submitted to Mechanical Stimulation. International Journal of Molecular Sciences 23 (2022) 10180.

[5] S. Dinarelli, G. Longo, G. Dietler, A. Francioso, L. Mosca, G. Pannitteri, G. Boumis, A. Bellelli, and M. Girasole, Erythrocyte’s aging in microgravity highlights how environmental stimuli shape metabolism and morphology. Scientific Reports 8 (2018) 5277.

[6] A. Saraswathibhatla, D. Indana, and O. Chaudhuri, Cell–extracellular matrix mechanotransduction in 3D. Nature Reviews Molecular Cell Biology 24 (2023) 495-516.

[7] N. Raman, S.A.M. Imran, K.B. Ahmad Amin Noordin, W. Zaman, and F. Nordin, Mechanotransduction in Mesenchymal Stem Cells (MSCs) Differentiation: A Review. Int J Mol Sci 23 (2022).

[8] K. Franze, P.A. Janmey, and J. Guck, Mechanics in neuronal development and repair. Annu Rev Biomed Eng 15 (2013) 227-51.

[9] C.A. Haselwandter, Y.R. Guo, Z. Fu, and R. MacKinnon, Quantitative prediction and measurement of Piezo's membrane footprint. Proceedings of the National Academy of Sciences of the United States of America 119 (2022) e2208027119.

[10] A.H. Lewis, M.E. Cronin, and J. Grandl, Piezo1 ion channels are capable of conformational signaling. Neuron 112 (2024) 3161-3175.e5.

[11] J. Wu, R. Goyal, and J. Grandl, Localized force application reveals mechanically sensitive domains of Piezo1. Nature communications 7 (2016) 12939.

[12] X. Wang, and T. Ha, Defining single molecular forces required to activate integrin and notch signaling. Science (New York, N.Y.) 340 (2013) 991-4.

[13] S. Morioka, T. Oishi, S. Hatazawa, T. Kakuta, T. Ogoshi, K. Umeda, N. Kodera, H. Kurumizaka, and M. Shibata, High-Speed Atomic Force Microscopy Reveals the Nucleosome Sliding and DNA Unwrapping/Wrapping Dynamics of Tail-less Nucleosomes. Nano letters 24 (2024) 5246-5254.

[14] M.H. Jo, J. Li, V. Jaumouillé, Y. Hao, J. Coppola, J. Yan, C.M. Waterman, T.A. Springer, and T. Ha, Single-molecule characterization of subtype-specific β1 integrin mechanics. Nature communications 13 (2022) 7471.

[15] J.E. Sader, Frequency response of cantilever beams immersed in viscous fluids with applications to the atomic force microscope. Journal of Applied Physics 84 (1998) 64-76.

[16] L.D. Landau, and E.M. Lifshitz, Fluid Mechanics: Volume 6, Elsevier, 1987.
